# ACE gene polymorphism and susceptibility to hypertension in a Jordanian adult population

**DOI:** 10.1371/journal.pone.0304271

**Published:** 2024-06-25

**Authors:** Laith AL-Eitan, Sara Al-Khaldi, Rasheed k. Ibdah

**Affiliations:** 1 Department of Biotechnology and Genetic Engineering, Jordan University of Science and Technology, Irbid, Jordan; 2 Department of Applied Biological Sciences, Jordan University of Science and Technology, Irbid, Jordan; 3 Internal Medicine Department, College of Medicine, Jordan University of Science and Technology, Irbid, Jordan; PearResearch / Government Doon Medical College, INDIA

## Abstract

Hypertension is one of the most common and complicated disorders associated with genetic and environmental risk factors. The angiotensin-converting enzyme (ACE) is important in the renin-angiotensin-system pathway. The gene expression of ACE has been investigated as a possible hypertension marker. This study investigates the association between polymorphisms within the ACE1 and ACE2 genes and hypertension susceptibility in a Jordanian population. The study comprised a total of 200 hypertensive patients and 180 healthy controls. A polymerase chain reaction (PCR) was performed to genotype the candidate polymorphism (rs4646994) of the ACE1gene. The Luminex DNA array technique was used for genotyping SNPs (rs4359, rs4344, rs4341, rs4343, and rs2106809) of the ACE1 and ACE2 genes. Our findings suggest no association between SNPs and hypertension regarding allelic and genotypic frequencies. However, rs4359 was significantly associated with diet (pP = 0.049), know HTN (P = 0.042), and number of years DM (P = 0.003). rs4341 was associated with diet (P = 0.032), peripheral vascular disease (P = 0.005), and chronic kidney disease (p = 0.049). While rs4343 was associated with diet (P = 0.031), diabetes mellitus (P = 0.032), and other medication (P = 0.025). Furthermore, the haplotypes of four SNPs of the ACE1 gene showed no significant association with HTN patients and healthy controls. Our findings indicate no association between the polymorphisms in the ACE gene and the risk of hypertension development in the Jordanian adult population.

## 1. Introduction

Hypertension (HTN) is characterized by elevated systolic and diastolic blood pressure [1]. It appears to result from a complicated interplay between genetic and environmental factors. Furthermore, it is a significant hazard factor for mortality and disability worldwide [2,3]. It is vastly associated with cardiovascular diseases (CVDs). In addition, it is implicated in substantially increasing the risk of stroke, cerebrovascular diseases, end-stage renal disease, organ damage, and other medical disorders [4–7].

The global prevalence of HTN was estimated to be 972 million in 2000. And it significantly rose to 1.3 billion in 2015 [8]. In Arab countries, it was estimated that 30 percent of adults suffered from HTN. [9]. Also, among Jordanians, one-third of adults suffer from HTN [10]. The risk factors that raise the risk of developing HTN can be divided into those that are modifiable, which are influenced by smoking, obesity, an excessive amount of salt, excessive alcohol consumption, and chronic stress. As well as non-modifiable ones, such as older age and family history [11]. Besides that, genetic risk factors account for approximately 30–50 percent of blood pressure variation [12].

Multiple studies have revealed that the renin-angiotensin system (RAS) has substantial direct participation in the regulation of blood pressure and is composed of several genes, such as renin, angiotensinogen (*AGT*), angiotensin II type 1 receptor (*AGTR1*), and angiotensin-1-converting enzyme (*ACE*) [13,14]. Angiotensin I-converting enzyme (ACE) is the main factor regulating blood pressure [15]. Also, it regulates the renin-angiotensin system (R*AS*) and the Kinin-Kallikrein system [16,17]. The *ACE* catalyzes the production of the vasoactive octapeptide (Ang II). Moreover, it inactivates the vasodilator bradykinin [18].

The human ACE gene spans approximately 21 kb and comprises 26 exons and 25 introns. It is located at location 23.3 on the long arm of chromosome 17 [19]. A specific variation in the *ACE* gene is called the *ACE* I/D polymorphism, which is determined by the 287 bp Alu repeat sequence’s presence (insertion) (I allele) or absence (deletion) (D allele) on ACE gene’s intron 16 [20]. In 1990, Rigat B et al. discovered the *ACE* gene polymorphism for the first time, and I/D polymorphism’s physiological significance was found through its association with plasma *ACE* levels, frequently studied in cardiovascular, hypertension, and other complex diseases [21].

Angiotensin II-converting enzyme (*ACE2)* was identified as the first reported *ACE* homolog in 2000 [22]. It promotes and induces vasodilation via the effective degradation of Ang II and the production of the vasodilator Angiotensin Ang 1–7 [23,24]. *ACE2* negatively regulates the renin-angiotensin system [33]—also the alternate arm of the RAS [25]. The *ACE*2 gene in humans is present along the short arm of Chromosome X, specifically position Xp22.2, spans approximately 39.98 kb of genomic DNA and contains 20 introns and 18 exons [26].

*ACE1* and its homolog *ACE2* are thus seen as effector regulators of blood pressure that have a counterbalancing role via the synthesis of vasoactive peptides in the renin-angiotensin system [27,28]. Meanwhile, disturbance of the tissue ACE/ACE2 homeostasis may result from functional ACE/ACE2 gene polymorphisms. Therefore, it could cause variations in blood pressure. Elevated blood pressure results from ACE2 deficiency, whereas higher ACE2 expression safeguards against elevated blood pressure [29]. Various previous studies have demonstrated and provided evidence that genetic variation in *ACE* genes (i.e., *ACE* I/D (rs4646994), G2350A (rs4343), (rs4341), (rs4344), (rs4359), (rs2106809)) is correlated with the pathogenesis of HTN [30–34]. Numerous gene families have been implicated in cardiovascular diseases, with multiple genetic variations spread across the Jordanian population. Research has also indicated that several SNPs may also influence the response to cardiovascular medications such as warfarin [35–44]. However, the relationship between polymorphism within the ACE gene and HTN is still debatable and has so far been inconclusive; the reason is ethnic variations and geographic features.

The study investigated the association between single nucleotide polymorphisms within the ACE1 and ACE2 genes and HTN susceptibility. Furthermore, it represents the first investigation into the association of rs4646994, rs4359, rs4344, rs4341, rs4343 in *ACE1*, and rs2106809 in *ACE2* gene with HTN risk in a Jordanian population.

## 2. Materials and methods

### 2.1. Study subject

The patient cohort analyzed in our study comprised individuals who regularly visit hypertension clinics within the Coronary Cardiac Care Department at King Abdullah University Hospital (KAUH) in Irbid, Jordan. The diagnosis of hypertension relied on the ICD-10 coding system, thus ensuring the inclusion of patients with confirmed hypertension. Given the regular attendance of these individuals at hypertension clinics and their continuous treatment regimen, further verification of the disease was considered unnecessary. Thus, our study collected blood samples and clinical records using the specified methodology. Patients enrolled in the case-control study were recruited from a single institutional hospital, and the sample size was restricted to the number of patients visiting the outpatient cardiac clinic and the Coronary Heart Care Department and recorded in the hospital.

In our case-control study, only 200 unrelated Jordanian patients were included after excluding patients who did not meet the inclusion criteria. The inclusion criteria necessitated official confirmation of hypertension diagnosis, data availability in the KAUH patient registry system, and participants aged 35 years or older. The patients who received various antihypertensive drugs were recruited from the outpatients of the cardiac clinic and the Coronary Heart Care Department during their follow-up visits at King Abdullah University Hospital (KAUH) and were classified into five groups based on the type of antihypertensive medication they were prescribed (ACEIs, ARBs, CCBs, BBs, and TDs), following the guidelines determined by the American Heart Association (AHA) and the Joint National Committee on the Detection, Evaluation, and Treatment of High Blood Pressure (JNC), as practiced at KAUH. The exclusion criteria included individuals who refused to provide written informed consent, those with insufficient clinical data, and individuals who were related up to the second degree with participants in the current study.

In addition, 180 unrelated adult healthy volunteers with no history of HTN were randomly chosen from the Jordanian population as controls. All research volunteers provided written informed consent. Ethical approval was received from the Institutional Review Board (IRB) committee at the Jordan University of Science and Technology with attached code No (4/133/2020) informed consent. All patients’ information was collected and analyzed, including patients’ medical records data, along with data on demographics (age, height, weight, and gender) and lifestyle (smoking status and diet).

According to the WHO, 1.3 million adults in Jordan between the ages of 30 and 79 have hypertension, with a prevalence of 12%, out of the country’s 10,699,000 total population (https://www.who.int/publications/m/item/hypertension-jor-2023-country-profile). The sample size was determined using the OpenEpi program, version 3.01, with a 95% confidence interval. Considering a prevalence of 12% of hypertension in Jordan, a precision of 5%, and a design effect 1, the sample size was calculated to be 163 patients. In our study, the sample size for the cases comprised 200 individuals. Initially, 320 patients were screened, but following the exclusion criteria, this number was reduced to 200, exceeding the requisite sample size.

### 2.2 General characteristics of HTN patients and healthy controls

In our study, 200 unrelated Jordanian patients were diagnosed with HTN and treated with various HTN medications. These patients included both males and females and the mean age ± SD was (58.84±10.39) years, with a range of (35–83) years. The control group had a mean age of (34.50±12.44), and the range was (18–80) years. The matched gender patients were selected with (58.4%) males and (41.6%) females, while the matched gender healthy controls were selected with (61.7%) males and (38.3%) females. Furthermore, all participants who met the inclusion criteria agreed and signed on to participate in our study. The patient’s and healthy control descriptions, including demographic data, are shown in Table 1.

**Table 1:** Description of the demographic characteristics of HTN patients and healthy controls.

Characteristics		Patients (n = 200)	Controls (n = 180)
**Gender**	**Male**	58.4%^a^	61.7%^a^
	**Female**	41.6%^a^	38.3%^a^
**Smoking**	**Yes**	27.9%^a^	44%^a^
	**No**	72.1%^a^	56%^a^
**Age (years)**		58.84±10.39^b^	34.50± 12.44^b^
**BMI(Kg/m** ^**2**^ **)**		33.35 ±22.58^b^	30.56±56.26^b^

^a^ Percentage %.

^b^ Mean ± Standard Deviation.

### 2.3 The candidate Gene and SNP selection

In our study, we selected six SNPs within candidate genes *ACE1* and *ACE2* that are specifically involved in susceptibility to HTN. The SNP database of the National Centre for Biotechnology Information (NCBI) (http://www.ncbi.nlm.nih.gov/SNP/) and the Ensemble database (http://www.ensembl.org/index.html) are two of the public databases from which these SNPs were selected. These SNPs were chosen based on their significant functional relevance, biological significance, and location within the selected gene. Furthermore, they are involved in the pathogenesis of HTN.

We further explored the functional annotations of the variants studied in this work using HaploReg v4.2 (https://pubs.broadinstitute.org/mammals/haploreg/haploreg.php) and RegulomeDB version 2.2 (https://regulomedb.org/regulome-search/).

The results derived from RegulomeDB showed that ACE rs4359 was graded 1F with a score of 0.55436, rs4344 was graded 1b with a score of 0.36991, rs4341 was graded 4 with a score of 0.60906, rs4343 was graded 2b with a score of 0.57391, and rs2106809 was graded 7 with a score of 0.51392, indicating that SNPs were likely to affect binding and be linked to the expression of a gene target. In addition, the functional annotation of HaploReg v4.2 showed that rs4359, rs4344, rs4341, rs4646994, and rs2106809, located in the intronic region of the ACE1 gene and ACE2, among them, rs4343 was a synonymous coding SNP, potentially affecting both promoter and enhancer histone marks and changing the transcription factor binding motifs. Consequently, these markers could indirectly contribute to the pathogenesis of hypertension by modulating the corresponding transcription factors of ACE.

### 2.4 DNA preparation

The QIAGEN Puregene® Blood Core kit-B extracted genomic DNA from frozen venous blood specimens according to the manufacturer’s instructions.

### 2.5 DNA analysis and genotyping

The Nano-Drop ND-1000 ultraviolet-visible spectrophotometer (Bio Drop, UK) was used to check the quality and quantity of isolated DNA. The polymerase chain reaction (PCR) method was applied for the analysis of the I/D polymorphism (rs4646994) of the *ACE1* gene using the primers flanking [38]. Gene amplification was also carried out using a PCR assay. The mixture was heated under the following conditions: initial denaturation at 94C° for 5 minutes, followed by 35 cycles of denaturation at 94C° for 30 seconds, annealing at 58 C° for 45 seconds, extension at 72C° for 30 seconds^,^ and final extension at 72C °for 2 minutes.

PCR products were visualized and separated by 3% agarose gel electrophoresis with ethidium bromide. The existence of 190 and 490 bp fragments allowed the identification of D or I alleles. Heterozygous D/I genotype show the existence of two bands at 190 bp and 490 bp. The Luminex DNA array technique was used for genotyping SNPs (rs4359, rs4344, rs4341, rs4343, and rs2106809) of the *ACE1* and *ACE2* genes in the Australian Genome Research Facility (AGRF; Melbourne Node, Melbourne, Australia).

Primers used in the study were chosen based on previously published scientific papers and derived from known human sequences. These are briefly summarized in Table 2.

**Table 2:** The sequences of forward and reverse primers of polymorphism in *ACE1* and *ACE2* genes.

SNP_ID	PCR Primer 1 (with 10mer Agena tag)	PCR Primer 2 (with 10mer Agena tag)
**rs4646994**	CTGGAGACCACTCCCATCCTTTCT	GAT GTG GCC ATC ACA TTC GTC AGA T
**rs4341**	ACGTTGGATGATTCTCTGAGCTCCCCTTAC	ACGTTGGATGATTCGTCAGATCTGGTAGGG
**rs4343**	ACGTTGGATGCCTACCAGATCTGACGAATG	ACGTTGGATGCATGCCCATAACAGGTCTTC
**rs4344**	ACGTTGGATGATGTTGTGATGGGTGCCGTG	ACGTTGGATGAGATCTATGTCGGGCAAGTC
**rs4359**	ACGTTGGATGAAGTTACAGTCACAGGCTGG	ACGTTGGATGGAAGCTCACAGAAATTGAGG
**rs2106809**	ACGTTGGATGGCTGCTGATGTAGAAGTGTG	ACGTTGGATGAGAAAGGGAGAGAACTTTGG

### 2.6 Gel electrophoresis and genotyping of *ACE1* gene

The genotypes of the SNP (rs4646994) in intron16 of the *ACE1* gene were analyzed using polymerase chain reaction (PCR) and gel electrophoresis, and the resulting genotypes were visualized on an agarose gel. In terms of band sizes, three genotypes were detected: a homozygous (D/D) genotype at 190 bp and a homozygous (I/I) genotype at 490 bp, with the presence of heterozygous alleles (D/I) indicated by the appearance of both bands 190 and 490 bp.

### 2.7 Statistical analysis

The data analysis used the Statistical Package for the Social Sciences (SPSS) version 25.0 (SPSS, Inc., Chicago, IL, USA). Pearson’s χ^2^-test and ANOVA were employed to perform genotype-phenotype analysis, assessing the normal distribution of variables, which were presented as Mean ± SD and statistically tested. Additionally, SNPStats, a web-based tool (https://www.snpstats.net/start.htm), was utilized to analyze single SNPs, including examination under multiple inheritance models and assess Hardy-Weinberg Equilibrium. Furthermore, SNPStats facilitated the analysis of various SNPs, allowing for the estimation of haplotype frequencies and the investigation of associations between haplotypes and disease conditions. The risk associated with genotypes/alleles was evaluated by calculating the Odds Ratio (OR) with a 95% confidence interval (CI). Statistical significance was determined at p<0.05. In examining genotype-to-genotype correlations, we utilized a post hoc multiple comparison test. Furthermore, multinomial logistic regression analysis was used to evaluate the potential influence of gene polymorphisms on the disease. We determined adequate SNPs using the reference [45] method for multiple testing corrections. The significance cut-off was also set at α/n, where α = 0.05 and n represents the number of tests, employing the Bonferroni correction [46].

## 3. Results

### 3.1 HWE test

All investigated SNPs met the criteria for HWE in both cases, and the controls were included in the study. One SNP (rs2106809) exhibited deviation from Hardy-Weinberg equilibrium (HWE) in both the control and patient groups, necessitating its exclusion from the analysis. In addition, SNP (rs4646994) demonstrated a deviation from HWE solely within the patient group. Notable, when the population deviates from the Hardy-Weinberg equilibrium, it means that particular mechanisms or processes drive evolution. Some variables might produce departures from HWE and upset the normal distribution, such as mutations, natural selection, gene flow, or the higher possibility of this divergence in our study, possibly due to non-random mating.

Table 3 displays the candidate genes with their associated polymorphisms. The distribution of each SNP’s minor allele in the cases and controls demonstrates standards-Weinberg equilibrium.

**Table 3:** A*CE1and ACE2* demonstrated SNPs, their minor allele frequencies, and HWE p-value in cases and controls.

Genes	SNP _ID	Cases (n = 200)			Controls (n = 180)		
		MA^a^	MAF^b^	HWE^c^*p-value*	MA^a^	MAF^b^	HWE^c^*p-value*
***ACE1***	rs4359	C	0.39	0.88	C	0.35	0.24
	rs4344	A	0.37	0.44	A	0.36	0.62
	rs4341	C	0.38	0.54	C	0.36	0.5
	rs4343	A	0.47	0.48	A	0.45	0.28
	rs4646994	I	0.7	**0.0045** *	I	0.69	0.58
***ACE2***	rs2106809	G	0.28	**<0.0001** *	G	0.16	**<0.0001** *

^a^ MA: Minor allele.

^b^ MAF: Minor allele frequency.

^c^ HWE: Hardy-Weinberg Equilibrium.

Significant P values are indicated by

* (P < 0.05).

### 3.2 Association of SNPs candidate genes with HTN

In our study, the association between HTN and candidate polymorphisms was investigated. No significant associations were found for all SNPs in the ACE1 gene, and neither the allelic nor genotypic frequencies between cases and controls showed any significant differences. As shown in Tables 4 and 5.

**Table 4:** Allele frequency of genes SNPs in HTN patients and controls.

Gene	SNP_ID	Allele/ Genotype	Controls (180)	Cases (200)	Controls vs. cases	
					chi-square	p-value
***ACE1***	**rs4359**	T	219(64.91%)	240(60.91%)	1.17	0.27
		C	119(3.215%)	154(39.09%)		
	**rs4344**	G	216(63.91%)	244(62.66%)	0.12	0.72
		A	122(36.09%)	146(37.34%)		
	**rs4341**	G	214(64.07%)	244(62.24%)	0.25	0.61
		C	120(35.93%)	148(37.76%)		
	**rs4343**	G	187(55.33%)	207(52.54%)	0.56	0.46
		A	151(44.67%)	187(47.67%)		

Significant P values are indicated by * (P< 0.05).

**Table 5:** Genotype frequency of genes SNPs in HTN patients and controls.

Gene	SNP_ID	Allele/ Genotype	Controls (180)	Cases (200)	Controls vs. cases	
					p-value	overall p-value
***ACE1***	**rs4359**	T/C	85(50.30%)	96(49%)	0.344	0.39
		C/C	17(10.06%)	29(14.72%)	0.767	
		T/T	67(39.64%)	72(36.55%)	0.244	
	**rs4344**	A/A	20(11.83%)	30(15.38%)	0.136	0.54
		G/G	67(39.64%)	79(40.51%)	0.555	
		A/G	82(48.52%)	86(44.10%)	0.3468	
	**rs4341**	G/G	66(%39.52)	78(39.82%)	0.461	0.50
		C/C	19(%11.83)	30(15.31%)	0.135	
		G/C	82(%49.10)	88(44.90%)	0.212	
	**rs4343**	G/G	48(28.40%)	57(28.93%)	0.461	0.29
		A/A	30(17.75%)	47(23.86%)	0.067	
		G/A	91(53.85%)	93(47.21%)	0.960	

This study used different genetic models in the genetic association analysis. Table 6 summarizes the genetic models (co-dominant, dominant, recessive, and over-dominant) and shows the chi-squared values between cases and healthy controls.

**Table 6:** Genetic models and distributions of SNPs within genes in HTN patients and control.

GENE	SNP_ID	Model	Cases%	Controls%	OR (95% CI)	p-value
***ACE1***	**rs4359**	T/T	72 (36.5%)	67 (39.6%)	1	0.39
		C/T	96 (48.7%)	85 (50.3%)	0.95 (0.61–1.48)	
		C/C	29 (14.7%)	17 (10.1%)	0.63 (0.32–1.25)	
		T/T	72 (36.5%)	67 (39.6%)	1	0.54
		C/T-C/C	125 (63.5%)	102 (60.4%)	0.88 (0.57–1.34)	
		T/T-C/T	168 (85.3%)	152 (89.9%)	1	0.18
		C/C	29 (14.7%)	17 (10.1%)	0.65 (0.34–1.23)	
		T/T-C/C	101 (51.3%)	84 (49.7%)	1	0.77
		C/T	96 (48.7%)	85 (50.3%)	1.06 (0.71–1.61)	
	**rs4344**	G/G	79 (40.5%)	67 (39.6%)	1	0.54
		G/A	86 (44.1%)	82 (48.5%)	1.12 (0.72–1.75)	
		A/A	30 (15.4%)	20 (11.8%)	0.79 (0.41–1.51)	
		G/G	79 (40.5%)	67 (39.6%)	1	0.87
		G/A-A/A	116 (59.5%)	102 (60.4%)	1.04 (0.68–1.58)	
		G/G-G/A	165 (84.6%)	149 (88.2%)	1	0.32
		A/A	30 (15.4%)	20 (11.8%)	0.74 (0.40–1.36)	
		G/G-A/A	109 (55.9%)	87 (51.5%)	1	0.4
		G/A	86 (44.1%)	82 (48.5%)	1.19 (0.79–1.81)	
	**rs4341**	G/G	78 (39.8%)	66 (39.5%)	1	0.5
		C/G	88 (44.9%)	82 (49.1%)	1.10 (0.71–1.72)	
		C/C	30 (15.3%)	19 (11.4%)	0.75 (0.39–1.45)	
		G/G	78 (39.8%)	66 (39.5%)	1	0.96
		C/G-C/C	118 (60.2%)	101 (60.5%)	1.01 (0.66–1.54)	
		G/G-C/G	166 (84.7%)	148 (88.6%)	1	0.27
		C/C	30 (15.3%)	19 (11.4%)	0.71 (0.38–1.32)	
		G/G-C/C	108 (55.1%)	85(50.9)	1	0.42
		C/G	88 (44.9%)	82 (49.1%)	1.18 (0.78–1.79)	
	**rs4343**	G/G	57 (28.9%)	48 (28.4%)	1	0.3
		A/G	93 (47.2%)	91 (53.9%)	1.16 (0.72–1.88)	
		A/A	47 (23.9%)	30 (17.8%)	0.76 (0.42–1.38)	
		G/G	57 (28.9%)	48 (28.4%)	1	0.91
		A/G-A/A	140 (71.1%)	121 (71.6%)	1.03 (0.65–1.62)	
		G/G-A/G	150 (76.1%)	139 (82.2%)	1	0.15
		A/A	47 (23.9%)	30 (17.8%)	0.69 (0.41–1.15)	
		G/G-A/A	104 (52.8%)	78 (46.1%)	1	0.21
		A/G	93 (47.2%)	91 (53.9%)	1.30 (0.86–1.97)	

P-values < 0.012 (0.05/# of SNPs, 0.05/4 = 0.012 after applying multiple comparison) are considered as comparisons) are consideredAnalysis of the ACE1 Gene.

For the SNPs (rs4359, rs4344, rs4343, and rs4341) in the *ACE1* gene, no genetic associations were found within all genetic models (co-dominant, dominant, recessive, and over-dominant) with HTN risk.

The haplotype was studied as a part of the genetic association analysis; our findings revealed the haplotypes of four SNPs on the promoter of the *ACE1* gene showed no significant association with HTN patients and healthy controls. As shown in Table 7.

**Table 7:** Frequencies of the haplotypes of genes among HTN patients and controls.

Gene	Haplotypes				Frequency		Odds Ratio (95% CI)	p-value
	rs4341	rs4343	rs4344	rs4359	Cases	Control		
***ACE1***	GGGT				0.5225	0.5497	1.00	-------
	CAAC				0.3652	0.343	0.87 (0.63–1.20)	0.39
	GAGT				0.0762	0.0803	0.98 (0.57–1.66)	0.93
	GAGC				0.0228	0.0055	0.22 (0.04–1.17)	0.076
	CAAT				0.0104	0.0179	1.52 (0.46–4.99)	0.49
	GGGC				0.0029	0.0035	1.06 (0.07–17.36)	0.97

### 3.4 Genotype versus phenotype association among HTN patients

The genotype-phenotype association between the SNPs (rs4344, rs4343, rs4359, and rs4341) of the *ACE1* gene and clinical characteristics related to HTN was determined by both Pearson’s chi-squared test and the ANOVA test. Accordingly, ACE1 rs4359 was significantly associated with diet (p = 0.049), know HTN (p = 0.042), and number of years DM (p = 0.003). rs4341 exhibited significantly associated with diet (p = 0.032), peripheral vascular disease (p = 0.005), and chronic kidney disease (p = 0.049). While rs4343 was associated with diet (p = 0.031), diabetes mellitus (p = 0.032), and other medication (p = 0.025). As shown in **S1 Table**.

### 3.5 Post hoc and regression analysis

In this research, regression analysis was utilized as a powerful stay robust method to explore the complex relationships between essential variables observed in the case and control groups. The Regression analysis sought to clarify the relationship between hypertension, symbolized by systolic and diastolic blood pressure measurements, and different clinical characteristics among the cases. By examining these relationships, we sought to identify possible factors influencing the onset and development of hypertension in our study sample. Moreover, our analysis expanded beyond simply exploration of the association between hypertension and clinical features. We also sought to clarify any possible relationship between particular genetic variants represented by the selected SNPs and the identified clinical features within the cases. This portion of the analysis allowed us to delve into the genetic foundations of hypertension and to identify any potential genetic markers related to particular clinical features of the condition. The comprehensive results of the regression analysis, which include the intricate relationships among hypertension, clinical features, and genetic variations, are presented in **S2, S3 and S4 Tables.** These tables present a comprehensive analysis of the statistical results and give essential details about the strength and direction of the correlations observed among the study cohort. Furthermore, to further explain the importance of our results, we performed post hoc analyses to compare individuals with different genotypes regarding clinical symptoms within the case group. Using these post hoc tests, we could determine statistically significant mean differences in variables among the cases, elucidating potential genotype-phenotype relationships and offering additional context to our regression analysis findings. The results described in **S5 Table** provide a critical addition to our regression analysis, providing additional information into the clinical implications of genetic variants linked to hypertension. By employing these rigorous analytical techniques, we attempt to thoroughly investigate hypertension’s complex, diverse nature, illuminating the complex interactions that arise between clinical features, genetic factors, and the development of this standard and intricate cardiovascular disease.

## 4. Discussion

Hypertension has been identified as a significant public health concern in the Middle East over several decades. Nevertheless, its impact exhibits pronounced heterogeneity, resulting in a widening health disparity between high-income and low to middle-income countries regarding various aspects, including awareness, diagnosis, treatment, and control [8]. The Middle East, a region known for its diversity and primarily situated in West Asia, exemplifies these discrepancies, with the Gulf countries predominantly constituting high-income nations. In contrast, others fall within the spectrum of low to middle-income status [47]. Moreover, this region is imbued with notable social, religious, and political variances, engendering substantial inequalities concerning access to healthcare, gender dynamics, daily lifestyles, and occupational opportunities [48]. Recent years have witnessed a swift urbanization process in the Middle East, concurrent with a heightened burden of chronic diseases, amidst national preventive health systems that have lagged mainly behind [49]. Given its relative ease of diagnosis, treatment, and management, hypertension presents a crucial focal point for scalable public health interventions in the region [50].

The prevalence of hypertension in the Middle East varies among countries and population groups. According to data from the Global Burden of Disease Study and other sources, hypertension prevalence in the Middle East has been steadily increasing. Based on a meta-analysis study in the Middle East, the overall prevalence of hypertension in the region was 24.36%. An increasing trend in the prevalence of hypertension was observed with increasing age [51]. Studies conducted in various Middle Eastern countries have reported high prevalence rates of hypertension among adults. In Saudi Arabia, for instance, hypertension has been found to affect between 26% to 30% of the adult population [52]. Similarly, research in Iran indicated a hypertension prevalence of approximately 25% among Iranian adults [53]. Moreover, studies in Jordan have reported that the age-standardized prevalence was 33.8% among men and 29.4% among women aged 18 to 90. [54].

HTN is a global health issue affecting over one billion adults and causing more than 9.4 million mortalities annually [55]. Only one in every four adults (24%) has HTN under control [56]. Despite the availability of numerous classes of effective drug options, the control rate remains uncontrolled and far from optimal [57]. It has been proven that the variants in the *ACE* genes affect the *ACE* plasma level and blood pressure regulation. Half, or 47%, of the overall phenotypic diversity of serum ACE levels is attributed to the insertion/deletion polymorphism [58]. The (I/D) polymorphism directly correlates to the diversity in plasma ACE levels; the (I) allele is related to diminished tissue concentrations of angiotensin II and a diminished enzyme level.

Meanwhile, D alleles have a high ACE plasma level, increased concentrations of angiotensin II, and degradation of bradykinin, which leads to hypertension [59–61]. Otherwise, in the linkage study, Ljungberg B et al., did not support that the A*CE* level is associated with HTN in both men and women, and there was no correlation between the *ACE* level and the systolic, diastolic or pulse pressure, which was not in agreement with most of the researchers [62]. Previous studies have been conducted in large populations to investigate the pivotal role of the angiotensin-converting enzyme *ACE* I/D polymorphism in hypertension in an Indian, a Sikh, and an African American population [63–65]. In contrast, our findings revealed that none of the SNPs in the *ACE1* gene were linked to HTN in all genetic models. as shown in Tables 4–6. In addition, the haplotype analysis revealed that the haplotypes of the ACE1 gene were not significantly associated with a risk of HTN in the Jordanian population. as shown in Table 7.

Following the HWE test for the investigated polymorphisms in the case and control sample sets, the genetic analysis for this study included all of the polymorphisms that satisfied the HWE criteria except for SNP (rs4646994) in the *ACE*1 in the case group *and* (rs2106809) in the *ACE2*. For that, any p-value less than (0.05) is considered not normally distributed, which would be for various reasons; the most likely is that the population size was not very large, making genetic drift negligible. Hardy-Weinberg equilibrium (HWE) tests are often used to rapidly examine genotype information, assuming that genotype frequencies ought to adhere to HWE proportions in a large population with random mating. Deviations from HWE, on the other hand, might result from a variety of different variables, such as purifying selection, copy number variation, inbreeding, or population-specific factors [66]; another possibility is that the Arabic population “including the Jordanian population,” tends to marry among relatives. Relative marriages have a significant influence on the distribution of genotypes in a population and have the potential to disturb HWE. Under normal circumstances, HWE assumes random mating in which individuals pick their spouses without regard for bias. However, when relatives marry or engage in consanguineous relationships, “non-random mating, “the genetic diversity within the population becomes restricted, resulting in a higher probability of shared alleles and observing that homozygous alleles are much more prevalent in that population [67].

When considering why one SNP might deviate from HWE from others in the same sample, there are several possibilities, including Random fluctuations in allele frequencies that can occur in small populations due to genetic drift. If a particular SNP is in a genomic region with low genetic diversity or subject to founder effects, it is more likely to deviate from equilibrium [68]. Natural selection can favor or reject specific alleles, causing deviations from HWE. If an SNP is under selection pressure due to its association with a beneficial or harmful trait, it may deviate from equilibrium [69]. New mutations can introduce novel alleles into a population. If an SNP experiences a recent mutation event or is in a region with a high mutation rate [70], it may deviate from HWE until the population reaches a new equilibrium. Deviations from HWE may also occur due to linkage disequilibrium, where a SNP is physically close to other genetic variants that influence their allele frequencies [71]. In such cases, deviations from equilibrium may be specific to certain SNPs due to their linkage with other genetic loci. These factors may explain the deviations from Hardy-Weinberg Equilibrium (HWE) for the particular SNP (rs2106809). To determine the type of deviations in rs2106809 SNP, we compare the observed genotype frequencies (AA, GA, GG) to the expected frequencies in the HWE equation (p^2^ + 2pq + q^2^ = 1). Based on this comparison, we observe an excess of individuals with the AA and GG genotypes compared to what is expected under HWE. In contrast, the observed frequencies of the GA genotype are lower than expected. The deviation observed in the genotype frequencies of the SNP (rs2106809) can be characterized as an excess of homozygotes (AA and GG genotypes) and a deficit of heterozygotes (GA genotype).

Additionally, the polymorphisms of the *ACE2* genes have been proven to affect and alter gene expression and are linked to the risk of disorders like left ventricular hypertrophy, dyslipidemia, and hypertension [72,73]. ACE2 polymorphisms have been identified as being correlated with hypertension, which is regarded as a prominent predictor of the severity of COVID-19 infection [74]. Blackwood EM, et al., in their study, clearly showed that the *ACE2* rs2106809 T allele might be functional, down-regulating *ACE2* expression and reducing promoter activity in the absence or presence of other SNPs, resulting in a decrease in the expression rate of circulating angiotensin (1–7) and contributing to an increase in blood pressure and an increased HTN susceptibility [75].

Our study observed that rs4343 in the ACE1 gene was not associated with HTN risk among Jordanians. And there are no significant differences in the allelic and genotypic frequencies between cases and controls. These results support the idea that the rs4343 SNP in the ACE1 gene would not contribute to HTN development among the Jordanian population. These findings agree with the results of other studies conducted with different populations. These results were consistent with an exciting study by Wang et al., who clearly showed no significant association with HTN risk in a total of 400 HTN patients compared with 100 healthy controls in patients in the Hefei Region, Anhui, China [76]. Besides that, these results agree with another study conducted in the Chinese Han population [77].

Conversely, however, specific studies of HTN in different populations find an association between the rs4343 polymorphism of ACE1 and HTN. In the Chinese Yi ethnic group, Yang et al. conducted a study using 244 HTN patients compared with 185 healthy controls and found an association between the rs4343 polymorphism of ACE1 and HTN susceptibility [78]. Similarly, in the Emirati population, the rs4343 polymorphism has been associated with an increased risk of essential HTN [79]. According to research by Jeong et al. on adult Koreans, the A allele of rs4343 raised the risk of hypertension by more than 2.1 fold, and high sodium intake enhanced this risk [80]. Comparably, Wang et al. found that rs4343 was a risk factor for high pulse pressure levels linked to hypertension and arterial elasticity [81].

The current study reported no significant association between rs4341 and rs4344 polymorphism and the risk of developing HTN. In contrast, a study in a Mexican population reported a significant association of rs4344 with the risk of developing HTN [82]. Meanwhile, rs4341 was associated with hypertension risk in the Pakistani population [83]. Limited studies have investigated the association of ACE polymorphism, rs4341, and rs4344 with HTN. The results of the current study need further investigation on a large sample group.

## 5. Conclusions

This study is the first to find the association of single nucleotide polymorphisms (SNPs) of the angiotensin-converting enzyme (ACE) genes with HTN risk in Jordan. It confirms and provides evidence that the ACE2 gene has no essential role in HTN susceptibility in the Jordanian population. This study further supports the gene-environment interaction model of HTN. Given the limited research on pharmacogenetics and personalized medicine in Jordan [84,85], this study requires further investigations into other Arab populations with many samples to support and confirm our findings.

## Supporting information

S1 Table Association between ACE1 SNP genotypes and the clinical characteristics of HTN. (XLSX)

S2 Table Regression analysis of the association between hypertension (systolic and diastolic) and clinical features. (XLSX)

S3 Table Regression analysis of the differences regarding demographic data, risk factors, and genetic variants between the case and control groups. (XLSX)

S4 Table Regression analysis of the differences regarding genetic variants and clinical features within the case group. (XLSX)

S5 Table Post hoc analysis of interactions between genetic variants and the clinical features of the case group. (XLSX)

## References

[1] Homan Travis D., Bordes Stephen, and Cichowski Erica. Physiology, pulse pressure. (2018). 29494015

[2] Joshi V, Lim J, Nandkumar M. Prevalence and risk factors of undetected elevated blood pressure in an elderly Southeast Asian population. Asia Pac J Public Health. 2007 Jun; 19(2):3–9. 10.1177/10105395070190020201 18050557

[3] Gharaibeh KA, Turner ST, Hamadah AM, Chapman AB, Cooper-Dehoff RM, Johnson JA, et al. Comparison of blood pressure control rates among recommended drug selection strategies for initial therapy of hypertension. Am J hypertension. 2016 Oct 1; 29(10):1186–94. 10.1093/ajh/hpw067 PMC5863787 27365079

[4] Olsen MH, Angell SY, Asma S, Boutouyrie P, Burger D, Chirinos JA, et al. A call to action and a lifecourse strategy to address the global burden of raised blood pressure on current and future generations: the Lancet Commission on hypertension. The Lancet. 2016 Nov 26; 388(10060):2665–712. 10.1016/S0140-6736(16)31134-5 27671667

[5] Coresh J, Wei GL, McQuillan G, Brancati FL, Levey AS, Jones C, et al. Prevalence of high blood pressure and elevated serum creatinine level in the United States: findings from the third National Health and Nutrition Examination Survey (1988–1994). Arch intern med. 2001 May 14; 161(9):1207–16. 11343443 10.1001/archinte.161.9.1207

[6] Arteriosclerosis A. report by the National Heart and Lung Institute Task Force on arteriosclerosis. Vol. II DHEW Pub. No (NIH). 1971:72–219.

[7] Messerli FH, Williams B, Ritz E. Essential hypertension. The Lancet. 2007 Aug 18; 370(9587):591–603. 10.1016/S0140-6736(07)61299-9 17707755

[8] Mills KT, Bundy JD, Kelly TN, Reed JE, Kearney PM, Reynolds K et al. Global disparities of hypertension prevalence and control: a systematic analysis of population-based studies from 90 countries. Circulation. 2016 Aug 9; 134(6):441–50. 27502908 10.1161/CIRCULATIONAHA.115.018912 PMC4979614

[9] Tailakh A, Evangelista LS, Mentes JC, Pike NA, Phillips LR, Morisky DE. Hypertension prevalence, awareness, and control in Arab countries: A systematic review. Nurs health sci. 2014 Mar; 16(1):126–30. 24118852 10.1111/nhs.12060 PMC4445843

[10] Khader Y, Batieha A, Jaddou H, Rawashdeh SI, El-Khateeb M, Hyassat D et al. Hypertension in Jordan: prevalence, awareness, control, and its associated factors. Int J Hypertension. 2019 May 2; 2019. 10.1155/2019/3210617 PMC6521462 31186953

[11] Morin DP, Bernard ML, Madias C, Rogers PA, Thihalolipavan S, Estes NM III. The state of the art: atrial fibrillation epidemiology, prevention, and treatment. In Mayo Clin Proc. 2016 Dec 1 91(12), 1778–1810. Elsevier. 27825618 10.1016/j.mayocp.2016.08.022

[12] Izawa H, Yamada Y, Okada T, Tanaka M, Hirayama H, Yokota M. Prediction of genetic risk for hypertension. Hypertension. 2003 May 1; 41(5):1035–40. 12654703 10.1161/01.HYP.0000065618.56368.24

[13] Sparks MA, Crowley SD, Gurley SB, Mirotsou M, Coffman TM. Classical renin-angiotensin system in kidney physiology. Compr Physiol. 2014 Jul; 4(3):1201. doi: 10.1002/cphy.c130040 24944035 PMC4137912

[14] Crowley SD, Coffman TM. Recent advances involving the renin–angiotensin system. Exp cell res. 2012 May 15; 318(9):1049–56. 22410251 10.1016/j.yexcr.2012.02.023 PMC3625040

[15] Zee RY, Lou YK, Griffiths LR, Morris BJ. Association of a polymorphism of the angiotensin I-converting enzyme gene with essential hypertension. Biochem biophys res commun. 1992 Apr 15; 184(1):9–15. 1314601 10.1016/0006-291x(92)91150-o

[16] Hikmet F, Méar L, Edvinsson Å, Micke P, Uhlén M, Lindskog C. The protein expression profile of ACE2 in human tissues. Mol syst biol. 2020 Jul; 16(7):e9610. 32715618 10.15252/msb.20209610 PMC7383091

[17] Jaspard E, Wei L, Alhenc-Gelas F. Differences in the properties and enzymatic specificities of the two active sites of angiotensin I-converting enzyme (kininase II). Studies with bradykinin and other natural peptides. J Biol Chem. 1993 May 5; 268(13): 9496–503. 7683654

[18] Corvol P, Eyries M, Soubrier F. Peptidyl-dipeptidase A/angiotensin I-converting enzyme. Handbook of proteolytic enzymes. Academic Press 2004 Jan 1:332–46.

[19] Baudin B. New Aspects on Angiotensin-Converting Enzyme: from Gene to Disease. 2002; 40(3): 256–265. 12005216 10.1515/CCLM.2002.042

[20] Ajala AR, Almeida SS, Rangel M, Palomino Z, Strufaldi MW, Puccini RF et al. Association of ACE gene insertion/deletion polymorphism with birth weight, blood pressure levels, and ACE activity in healthy children. 2012; 827–832 doi: 10.1038/ajh.2012.50 22647781

[21] Rigat B, Hubert C, Alhenc-Gelas F, Cambien F, Corvol P, Soubrier F. An insertion/deletion polymorphism in the angiotensin I-converting enzyme gene accounting for half the variance of serum enzyme levels. J clin investig. 1990 Oct 1; 86(4): 1343–6. doi: 10.1172/JCI114844 1976655 PMC296868

[22] Tipnis SR, Hooper NM, Hyde R, Karran E, Christie G, Turner AJ. A human homolog of angiotensin-converting enzyme: cloning and functional expression as a captopril-insensitive carboxypeptidase. J Biol Chem. 2000 Oct 27; 275(43): 33238–43. 10924499 10.1074/jbc.M002615200

[23] Warner FJ, Smith AI, Hooper NM, Turner AJ. Angiotensin-converting enzyme-2: a molecular and cellular perspective. Cell mol life sci: CMLS. 2004 Nov 1; 61(21):2704–13. doi: 10.1007/s00018-004-4240-7 15549171 PMC7079784

[24] Vickers C. Hales P, Kaushik V, Dick L, Gavin J, Tang J et al. Hydrolysis of biological peptides by human angiotensin-converting enzyme-related carboxypeptidase. J Biol Chem. 2002; 277:14838–43. doi: 10.1074/jbc.M200581200 11815627

[25] Patel SK, Velkoska E, Freeman M, Wai B, Lancefield TF, Burrell LM. From gene to protein—experimental and clinical studies of ACE2 in blood pressure control and arterial hypertension. Front in physiol. 2014 Jun 24; 5:227. doi: 10.3389/fphys.2014.00227 25009501 PMC4067757

[26] Samavati L, Uhal BD. ACE2, much more than just a receptor for SARS-COV-2. Front cell and infect microbiol. 2020:317. doi: 10.3389/fcimb.2020.00317 32582574 PMC7294848

[27] Luo Y, Liu C, Guan T, Li Y, Lai Y, Li F et al. Association of ACE2 genetic polymorphisms with hypertension-related target organ damages in south Xinjiang. Hypertension Research. 2019 May; 42(5):681–9. doi: 10.1038/s41440-018-0166-6 30542083 PMC6477792

[28] Reddy Gaddam R, Chambers S, Bhatia M. ACE and ACE2 in inflammation: a tale of two enzymes. Inflamm. Allergy-Drug Targets (Former. Curr. Drug Targets—Inflamm. Allergy) (Discontin.). 2014 Aug 1; 13(4):224–34. 10.2174/1871528113666140713164506 25019157

[29] Yagil Y, Yagil C. Hypothesis: ACE2 modulates blood pressure in the mammalian organism. Hypertension. 2003 Apr 1; 41(4):871–3. doi: 10.1161/01.HYP.0000063886.71596.C8 12654716

[30] Li Y. Angiotensin‐converting enzyme gene insertion/deletion polymorphism and essential hypertension in the Chinese population: a meta‐analysis including 21 058 participants. Intern med J. 2012 Apr; 42(4):439–44. doi: 10.1111/j.1445-5994.2011.02584.x 21883781

[31] Niu W, Qi Y, Hou S, Zhai X, Zhou W, Qiu C. Haplotype-based association of the renin-angiotensin-aldosterone system genes polymorphisms with essential hypertension among Han Chinese: the Fangshan study. J hypertension. 2009 Jul 1; 27(7):1384–91. doi: 10.1097/HJH.0b013e32832b7e0d 19412130

[32] Eisenmann JC, Sarzynski MA, Glenn K, Rothschild M, Heelan KA. ACE I/D genotype, adiposity, and blood pressure in children. Cardiovasc Diabetol. 2009 Dec; 8(1):1–8. doi: 10.1186/1475-2840-8-14 19291311 PMC2658665

[33] Bhatnagar V, O’Connor DT, Schork NJ, Salem RM, Nievergelt CM, Rana BKet al. Angiotensin-converting enzyme gene polymorphism predicts the time-course of blood pressure response to angiotensin converting enzyme inhibition in the AASK trial. J hypertension. 2007 Oct; 25(10):2082. doi: 10.1097/HJH.0b013e3282b9720e 17885551 PMC2792638

[34] Pan Y, Wang T, Li Y, Guan T, Lai Y, Shen Y et al. Association of ACE2 polymorphisms with susceptibility to essential hypertension and dyslipidemia in Xinjiang, China. Lipids in health and disease. 2018 Dec; 17(1):1–9. 30342552 10.1186/s12944-018-0890-6 PMC6195726

[35] Alghamdi M.A., et al., Variants in CDHR3, CACNAC1, and LTA Genes Predisposing Sensitivity and Response to Warfarin in Patients with Cardiovascular Disease. International Journal of General Medicine, 2021: p. 1093–1100. doi: 10.2147/IJGM.S298597 33790638 PMC8006967

[36] Al-Eitan L.N., Almasri A.Y., and Khasawneh R.H., Effects of CYP2C9 and VKORC1 polymorphisms on warfarin sensitivity and responsiveness during the stabilization phase of therapy. Saudi Pharmaceutical Journal, 2019. 27(4): p. 484–490. doi: 10.1016/j.jsps.2019.01.011 31061616 PMC6488816

[37] Al-Eitan L.N., Almasri A.Y., and Al-Habahbeh S.O., Impact of a variable number tandem repeat in the CYP2C9 promoter on warfarin sensitivity and responsiveness in Jordanians with cardiovascular disease. Pharmacogenomics and Personalized Medicine, 2019: p. 15–22. doi: 10.2147/PGPM.S189838 30962704 PMC6432888

[38] Al-Eitan L.N., Almasri A.Y., and Al-Habahbeh S.O., Effects of coagulation factor VII polymorphisms on warfarin sensitivity and responsiveness in Jordanian cardiovascular patients during the initiation and maintenance phases of warfarin therapy. Pharmacogenomics and Personalized Medicine, 2019: p. 1–8. doi: 10.2147/PGPM.S189458 30679919 PMC6338106

[39] Al-Eitan L.N., et al., Influence of SH2B3, MTHFD1L, GGCX, and ITGB3 Gene Polymorphisms on theVariability on Warfarin Dosage Requirements and Susceptibility to CVD in the Jordanian Population. Journal of Personalized Medicine, 2020. 10(3): p. 117. doi: 10.3390/jpm10030117 32916786 PMC7564501

[40] Al-Eitan L.N., Almasri A.Y., and Khasawneh R.H., Impact of CYP2C9 and VKORC1 polymorphisms on warfarin sensitivity and responsiveness in Jordanian cardiovascular patients during the initiation therapy. Genes, 2018. 9(12): p. 578. doi: 10.3390/genes9120578 30486437 PMC6316567

[41] Al-Eitan L.N., et al., Influence of CYP4F2, ApoE, and CYP2A6 gene polymorphisms on the variability of Warfarin dosage requirements and susceptibility to cardiovascular disease in Jordan. International journal of medical sciences, 2021. 18(3): p. 826. doi: 10.7150/ijms.51546 33437219 PMC7797549

[42] Al-Eitan L.N., et al., Influence of PSRC1, CELSR2, and SORT1 gene polymorphisms on the variability of warfarin dosage and susceptibility to cardiovascular disease. Pharmacogenomics and Personalized Medicine, 2020: p. 619–632. doi: 10.2147/PGPM.S274246 33235484 PMC7680183

[43] Al-Eitan L. N., Almasri A. Y., Alnaamneh A. H., & Mihyar A. Effect of MEF2A and SLC22A3-LPAL2-LPA gene polymorphisms on warfarin sensitivity and responsiveness in Jordanian cardiovascular patients. PloS one, (2023). 18(11), e0294226. doi: 10.1371/journal.pone.0294226 37948393 PMC10637663

[44] Ibdah R.K., et al., Impact of PCSK9, WDR12, CDKN2A, and CXCL12 polymorphisms in Jordanian cardiovascular patients on warfarin responsiveness and sensitivity. International Journal of General Medicine, 2021: p. 103–118. doi: 10.2147/IJGM.S287238 33488114 PMC7814275

[45] Nyholt DR. A simple correction for multiple testing for single-nucleotide polymorphisms in linkage disequilibrium with each other. Am J Hum Genet, 2004. 74(4): p. 765–9. doi: 10.1086/383251 14997420 PMC1181954

[46] Li J, Ji L. Adjusting multiple testing in multilocus analyses using the eigenvalues of a correlation matrix. Heredity. 2005. 95(3): p. 221–7. doi: 10.1038/sj.hdy.6800717 16077740

[47] UN. World Economic Situation and Prospects 2020. New York: UN; 2020.

[48] Krishnan NLI, Gabriel; Narayan Ambar; Tiwari Sailesh; Vishwanath Tara. 2016. Uneven Odds, Unequal Outcomes: Inequality of Opportunity in the Middle East and North Africa. Directions in Development-Poverty;. Washington, DC: World Bank. https://openknowledge.worldbank.org/handle/10986/24596

[49] Alsheikh-Ali AA, Omar MI, Raal FJ, Rashed W, Hamoui O, Kane A, et al. Cardiovascular risk factor burden in Africa and the Middle East: the Africa Middle East Cardiovascular Epidemiological (ACE) study. PLoS One 2014;9:e102830. doi: 10.1371/journal.pone.0102830 25090638 PMC4121128

[50] Yusufali AM, Khatib R, Islam S, Alhabib KF, Bahonar A, Swidan HM, et al. Prevalence, awareness, treatment and control of hypertension in four Middle East countries. J Hypertens. 2017;35:1457–64. doi: 10.1097/HJH.0000000000001326 28486270

[51] Okati-Aliabad H, Ansari-Moghaddam A, Kargar S, Mohammadi M. Prevalence of hypertension and pre-hypertension in the Middle East region: a systematic review & meta-analysis. J Hum Hypertens. 2022 Sep;36(9):794–804. doi: 10.1038/s41371-021-00647-9 Epub 2022 Jan 15. 35031669

[52] Al-Nozha MM, Abdullah M, Arafah MR, et al. Hypertension in Saudi Arabia. Saudi Med J. 2007;28(1), 77–84. 17206295

[53] Haghdoost AA, Sadeghirad B, Rezazadehkermani M. Epidemiology and heterogeneity of hypertension in Iran: a systematic review. Arch Iran Med. 2008;11(4), 444–452. 18588378

[54] Khader Y, Batieha A, Jaddou H, Rawashdeh SI, El-Khateeb M, Hyassat D, et al. Hypertension in Jordan: Prevalence, Awareness, Control, and Its Associated Factors. Int J Hypertens. 2019 May 2;2019:3210617 31186953 10.1155/2019/3210617 PMC6521462

[55] Mills KT, Stefanescu A, He J. The global epidemiology of hypertension. Nat Rev Nephrol. 2020 Apr; 16(4):223–37. doi: 10.1038/s41581-019-0244-2 32024986 PMC7998524

[56] Centers for Disease Control and Prevention (CDC). Hypertension cascade: hypertension prevalence, treatment and control estimates among US adults aged 18 years and older applying the criteria from the American College of Cardiology and American Heart Association’s 2017 Hypertension Guideline—NHANES 2013–2016. Atlanta, GA: US Department of Health and Human Services. 2019.

[57] Calhoun D.A., Jones D., Textor S., Goff D.C., Murphy T.P., Toto R.D. et al. Resistant hypertension: diagnosis, evaluation, and treatment: a scientific statement from the American Heart Association Professional Education Committee of the Council for High Blood Pressure Research. Hypertension. 2008; 51(6):1403–1419. 18391085 10.1161/HYPERTENSIONAHA.108.189141

[58] Rigat B, Hubert C, Alhenc-Gelas F, Cambien F, Corvol P, Soubrier F. An insertion/deletion polymorphism in the angiotensin I-converting enzyme gene accounting for half the variance of serum enzyme levels. J clin investig. 1990 Oct 1;86(4):1343–6. doi: 10.1172/JCI114844 1976655 PMC296868

[59] Sakuma T, Hirata RD, Hirata MH. Five polymorphisms in gene candidates for cardiovascular disease in Afro‐Brazilian individuals. J clin lab anal. 2004; 18(6):309–16. doi: 10.1002/jcla.20044 15543563 PMC6807947

[60] Gainer JV, Nadeau JH, Ryder D, Brown NJ. Increased sensitivity to bradykinin among African Americans. J allergy clin immunol. 1996 Aug 1; 98(2): 283–7. doi: 10.1016/s0091-6749(96)70151-3 8757204

[61] Brown NJ, BlaisJr C, Gandhi SK, Adam A. ACE insertion/deletion genotype affects bradykinin metabolism. J cardiovasc pharmacol. 1998 Sep 1; 32(3): 373–7. doi: 10.1097/00005344-199809000-00006 9733349

[62] Ljungberg L, Alehagen U, Länne T, Björck H, De Basso R, Dahlström U et al. The association between circulating angiotensin-converting enzyme and cardiovascular risk in the elderly: a cross-sectional study. J Renin-Angiotensin-Aldosterone Syst. 2011 Sep; 12(3):281–9. doi: 10.1177/1470320310391326 21273224

[63] Borah PK, Shankarishan P, Hazarika NC, Mahanta J. Hypertension subtypes and angiotensin converting enzyme (ACE) gene polymorphism in Indian population. JAPI. 2012 Jun 1; 60(11):7–15. 23409414

[64] Mastana S, Nunn J. Angiotensin-converting enzyme deletion polymorphism is associated with hypertension in a Sikh population. Hum hered. 1997; 47(5):250–3. doi: 10.1159/000154420 9358012

[65] Bhatnagar V, O’Connor DT, Schork NJ, Salem RM, Nievergelt CM, Rana BK et al. Angiotensin-converting enzyme gene polymorphism predicts the time-course of blood pressure response to angiotensin converting enzyme inhibition in the AASK trial. Journal of hypertension. 2007 Oct; 25(10):2082. doi: 10.1097/HJH.0b013e3282b9720e 17885551 PMC2792638

[66] Chen B., Cole J. W., & Grond-Ginsbach C. Departure from Hardy Weinberg Equilibrium and Genotyping Error. Front genet. 2017; 8, 167. doi: 10.3389/fgene.2017.00167 29163635 PMC5671567

[67] abehji M. Hardy-Weinberg Equilibrium in the Large Scale Genomic Sequencing Era. Front genet. 2020; 11, 210. doi: 10.3389/fgene.2020.00210 32231685 PMC7083100

[68] Kimura M, Ohta T. Distribution of allelic frequencies in a finite population under stepwise production of neutral alleles. Proc Natl Acad Sci U S A. 1975 Jul;72(7):2761–4. doi: 10.1073/pnas.72.7.2761 1058491 PMC432851

[69] Nielsen R. Molecular signatures of natural selection. Annu Rev Genet. 2005;39:197–218. doi: 10.1146/annurev.genet.39.073003.112420 16285858

[70] Drake JW, Charlesworth B, Charlesworth D, Crow JF. Rates of spontaneous mutation. Genetics. 1998 Apr;148(4):1667–86. doi: 10.1093/genetics/148.4.1667 9560386 PMC1460098

[71] Slatkin M. Linkage disequilibrium—understanding the evolutionary past and mapping the medical future. Nat Rev Genet. 2008 Jun;9(6):477–85. doi: 10.1038/nrg2361 18427557 PMC5124487

[72] Fan Z, Wu G, Yue M, Ye J, Chen Y, Xu B et al. Hypertension and hypertensive left ventricular hypertrophy are associated with ACE2 genetic polymorphism. Life sci. 2019 May 15; 225:39–45. doi: 10.1016/j.lfs.2019.03.059 30917908

[73] Medina-Enríquez MM, Carlos-Escalante JA, Aponte-Torres Z, Cuapio A, Lopez-Leon S, Wegman-Ostrosky T. ACE2: the molecular doorway to SARS-CoV-2. Cell biosci. 2020; 10.1: 1–17. 33380340 10.1186/s13578-020-00519-8 PMC7772801

[74] Abdelsattar S, Kasemy ZA, Ewida SF, Abo-Elsoud RA, Zytoon AA, Abdelaal GA, Abdelgawad AS, Khalil FO, Kamel HF. ACE2 and TMPRSS2 SNPs as Determinants of Susceptibility to, and Severity of, a COVID-19 Infection. Br J Biomed Sci. 2022 Jan 12;79:10238. doi: 10.3389/bjbs.2021.10238 35996506 PMC8915702

[75] Blackwood EM, Kadonaga JT. Going the distance: a current view of enhancer action. Science. 1998 Jul 3; 281(5373):60–3. doi: 10.1126/science.281.5373.60 9679020

[76] Wang L, Song TT, Dong CW. Association between Interactions among ACE Gene Polymorphisms and Essential Hypertension in Patients in the Hefei Region, Anhui, China. J Renin Angiotensin Aldosterone Syst. 2023 Apr 13; 1159973. doi: 10.1155/2023/1159973 37091860 PMC10118893

[77] Pan M, Zhu JH, Liu ZH, Jiang WP, Cui ZC, Yu XH, et al. Angiotensin-converting enzyme gene 2350 G/A polymorphism is associated with left ventricular hypertrophy but not essential hypertension. Hypertens Res. 2007 Jan;30(1):31–7. doi: 10.1291/hypres.30.31 17460369

[78] Yang YL, Mo YP, He YS, Yang F, Xu Y, Li CC, et al. Correlation between renin-angiotensin system gene polymorphisms and essential hypertension in the Chinese Yi ethnic group. J Renin Angiotensin Aldosterone Syst. 2015 Dec;16(4):975–81. doi: 10.1177/1470320315598697 26283679

[79] Saeed Mahmood M, Saboohi K, Osman Ali S, Bokhari AM, Frossard PM. Association of the angiotensin-converting enzyme (ACE) gene G2350A dimorphism with essential hypertension. J Hum Hypertens. 2003 Oct;17(10):719–23. doi: 10.1038/sj.jhh.1001600 14504631

[80] Jeong S, Kim JY, Cho Y, Koh SB, Kim N, Choi JR. Genetically, dietary sodium intake is causally associated with salt-sensitive hypertension risk in a community-based cohort study: a Mendelian randomization approach. *Curr Hypertens Rep*. (2020) 22:45. doi: 10.1007/s11906-020-01050-4 32591971

[81] Wang N, Li X, Zhang Q, Zhang H, Zhou L, Wu N, et al. Association of angiotensin-converting enzyme gene polymorphism with pulse pressure and its interaction with obesity status in Heilongjiang province. *Clin Exp Hypertens*. (2018) 41:70–4. doi: 10.1080/10641963.2018.1445749 29546999

[82] Martínez-Rodríguez N, Posadas-Romero C, Villarreal-Molina T, Vallejo M, Del-Valle-Mondragón L, Ramírez-Bello J, et al. Single nucleotide polymorphisms of the angiotensin-converting enzyme (ACE) gene are associated with essential hypertension and increased ACE enzyme levels in Mexican individuals. PLoS One. 2013 May 31;8(5):e65700. doi: 10.1371/journal.pone.0065700 23741507 PMC3669228

[83] Shahid S. U., Cooper J. A., Rehman A., & Humphries S. E. Association of ACE and NOS3 gene polymorphisms with blood pressure in a case control study of coronary artery disease in Punjab, Pakistan. Pakistan Journal of Zoology, 2016. 48(4): 1125–1132.

[84] Al-Eitan L.N., and Haddad Y.A., Emergence of pharmacogenomics in academic medicine and public health in Jordan: history, present state and prospects. Current Pharmacogenomics and Personalized Medicine (Formerly Current Pharmacogenomics), 2014. 12(3): p. 167–175.

[85] Al-Eitan L.N., and Tarkhan A.H., Practical challenges and translational issues in pharmacogenomics and personalized medicine from 2010 onwards. Current Pharmacogenomics and Personalized Medicine (Formerly Current Pharmacogenomics), 2016. 14(1): p. 7–17.

